# Ex Vivo Preservation of Ovine Periosteum Using a Perfusion Bioreactor System

**DOI:** 10.3390/cells12131724

**Published:** 2023-06-27

**Authors:** Hai Xin, Sara Romanazzo, Eva Tomaskovic-Crook, Timothy C. Mitchell, Jui Chien Hung, Steven G. Wise, Kai Cheng, D S Abdullah Al Maruf, Murray J. Stokan, Timothy G. H. Manzie, Krishnan Parthasarathi, Veronica K. Y. Cheung, Ruta Gupta, Mark Ly, Carlo Pulitano, Innes K. Wise, Jeremy M. Crook, Jonathan R. Clark

**Affiliations:** 1 Integrated Prosthetics and Reconstruction, Department of Head and Neck Surgery, Chris O’Brien Lifehouse, Camperdown, NSW 2050, Australia; 2 Central Clinical School, Faculty of Medicine and Health, The University of Sydney, Camperdown, NSW 2050, Australia; 3 Arto Hardy Family Biomedical Innovation Hub, Chris O’Brien Lifehouse, Camperdown, NSW 2050, Australia; 4 School of Medical Sciences, Faculty of Medicine and Health, The University of Sydney, Sydney, NSW 2006, Australia; 5 Intelligent Polymer Research Institute, University of Wollongong, Innovation Campus, Squires Way, North Wollongong, NSW 2500, Australia; 6 Royal Prince Alfred Institute of Academic Surgery, Royal Prince Alfred Hospital, Sydney Local Health District, Camperdown, NSW 2050, Australia; 7 The Department of Tissue Pathology and Diagnostic Oncology, Royal Prince Alfred Hospital, Camperdown, NSW 2050, Australia; 8 RPA Translational Center for Organ Assessment, Repair, and Optimization, Royal Prince Alfred Hospital, Camperdown, NSW 2050, Australia; 9 Laboratory Animal Services, Charles Perkins Center, The University of Sydney, Camperdown, NSW 2050, Australia

**Keywords:** periosteum, perfusion, viability, bone engineering, transplantation, bone repair

## Abstract

Periosteum is a highly vascularized membrane lining the surface of bones. It plays essential roles in bone repair following injury and reconstruction following invasive surgeries. To broaden the use of periosteum, including for augmenting in vitro bone engineering and/or in vivo bone repair, we have developed an ex vivo perfusion bioreactor system to maintain the cellular viability and metabolism of surgically resected periosteal flaps. Each specimen was placed in a 3D printed bioreactor connected to a peristaltic pump designed for the optimal flow rates of tissue perfusate. Nutrients and oxygen were perfused via the periosteal arteries to mimic physiological conditions. Biochemical assays and histological staining indicate component cell viability after perfusion for almost 4 weeks. Our work provides the proof-of-concept of ex vivo periosteum perfusion for long-term tissue preservation, paving the way for innovative bone engineering approaches that use autotransplanted periosteum to enhance in vivo bone repair.

## 1. Introduction

Critical sized bone defects following trauma or surgical excision are traditionally treated using autologous or allogeneic bone grafts or vascularised bone flaps [1,2,3]. Bone tissue engineering represents a more contemporary approach that employs customizable scaffolds to recreate complex anatomy incorporating osteogenic materials and cells to stimulate new bone formation. It has great potential to reduce the morbidity associated with current treatment methods such as autologous bone reconstruction [4,5,6,7,8]. A critical challenge for tissue-engineered bone is that of vascularize the synthetic bone so it can self-heal and survive within the hostile environment of, for example, the irradiated oral cavity. One solution is to encase it within vascularized tissue, such as periosteum, which contains osteogenic progenitor cells and growth factors that are imperative for osteogenesis and angiogenesis [9,10].

Periosteum is a thin film covering the bone. It consists of two layers: a fibrous outer layer and a cell-rich inner cambium layer that contains osteoprogenitor cells, and osteoblasts [9,11,12,13]. As such, the periosteum is a major contributor to bone development, growth, and fracture healing. Clinical practices involving the use of periosteum have been widely reported, including midface reconstruction, periodontal furcation repair, and alveolar ridge augmentation where the periosteum enhances new bone formation and vascularization [14,15,16,17]. The use of periosteum for promoting the growth of maxillofacial bone within scaffold constructs containing osteogenic materials such as hydroxyapatite or tricalcium phosphate is well described [4,18,19,20,21]. Research includes wrapping a bioreactor scaffold with periosteum to vascularize the construct and induce bone formation [22,23,24]. Given the clinical utility of periosteum for bone engineering and reconstruction, ex vivo periosteal preservation could open up additional opportunities whereby vascularized periosteal tissue (i.e., a free flap) could be combined with a customized bone scaffold to induce new bone growth and vascularization in vitro ahead of autotransplantation for bone repair. 

Here, we report ex vivo periosteal preservation using a perfusion bioreactor system designed to provide tissue-specific physiological conditions where the oxygen and nutrients were perfused via their vascular network to maintain tissue viability. A peristaltic pump was used to circulate perfusate through periosteal explants maintained in a 3D printed bioreactor housed within a sterile, 5% CO_2_ humidified incubator at 37 °C. The real-time monitoring of the functional capacity of explants included the periodic collection of tissue biopsies and culture medium for biochemical, histological, and pH characterization, as well as the post-perfusion analysis of tissues to provide a comprehensive picture of their microanatomy. Our best experimental results demonstrate the viability of periosteal tissue for up to 25 days. To our knowledge, this is the first report of periosteal preservation in a perfusion bioreactor system ex vivo, with the goal of innovating bone engineering and in vivo bone repair.

## 2. Materials and Methods

### 2.1. Periosteum Procurement

**Figure 1:**
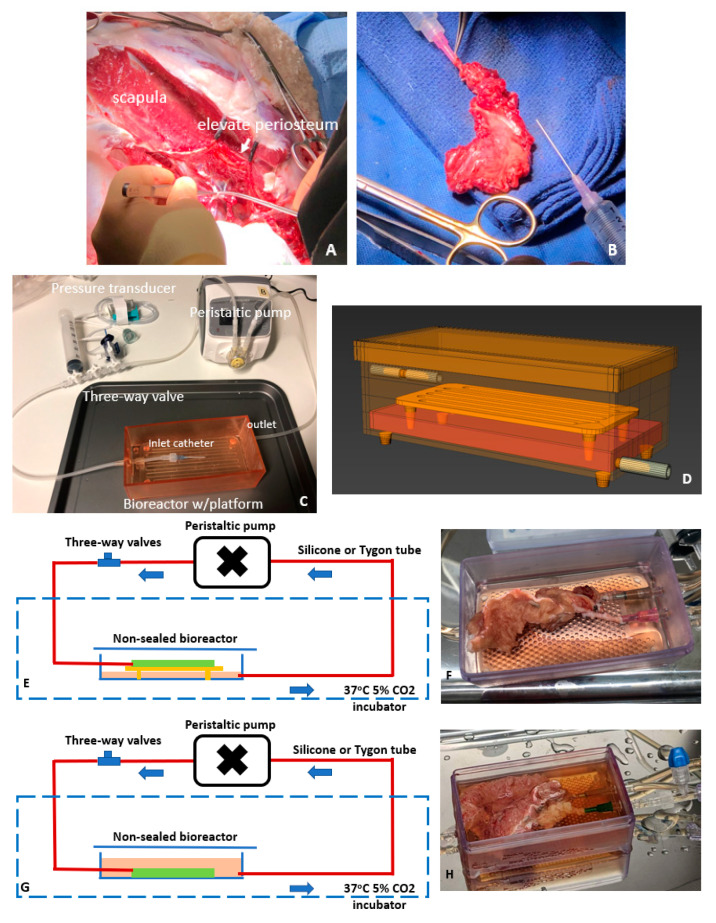
Periosteum procurement and ex vivo perfusion for tissue preservation. Vascularized periosteal flaps collected from sheep scapula (**A**,**B**). Primary components of the perfusion bioreactor system for periosteal maintenance (**C**,**D**). Perfusion system configuration for dry perfusion (**E**,**F**) and wet perfusion (**G**,**H**).

Six periosteal flaps were obtained from three female Dorset-cross sheep (aged 6–8 years) cared for by Laboratory Animal Services at Charles Perkins Center, The University of Sydney. Animals included in this study were approved by the University of Sydney Animal Ethics Committee under the following institutional research approval (2021/2004). As the tissues were acquired post mortem, additional approval for this study was not required. Following euthanasia, the periosteal flaps were immediately procured to minimize the ischemic period. Two periostea were recovered from the femur and weighed between 7 and 8 g while four periostea were procured from the scapula, weighing between 18 and 21 g. Each periosteal flap contained an appropriate amount of fat and muscular tissues to maintain the periosteal vascular network. As shown in Figure 1A,B, each sample was perfused with heparinized saline immediately after its procurement to remove residual blood, followed by the insertion and suturing of a perfusion catheter (Terumo Corporation, Tokyo, Japan) into the periosteal artery. Depending on the artery size, either 18G or 20G catheter was used. Periostea were subsequently placed in saline at 4 °C and immediately transported on ice to a PC2 laboratory at the Arto Hardy Family Biomedical Innovation Hub at Chris O’Brien Lifehouse.

### 2.2. Perfusion Systems and Experimental Regimen for Testing Periosteal Perfusion

The periosteal perfusion bioreactor system consisted of a 3D printed bioreactor, a peristaltic pump, connection tubing, incubator, and culture medium (Figure 1C,D). The peristaltic pump was connected via the bioreactor to the arterial catheter to circulate the perfusate though the periosteum vasculature. The bioreactor was 3D printed with a Form3 stereolithography printer using Surgical Resin V1 (Formlabs, Somerville, MA, USA). The inlet port was connected to the catheter while the bioreactor outlet at the bottom extracted the spent perfusate from the venous outflow, returning the perfusate to the pump to form a complete perfusion loop. The bioreactor was placed in a sterile, 5% CO_2_ humidified incubator at 37 °C (NuAire, Plymouth, MN, USA), while the pump was placed next to the incubator at room temperature. A pressure transducer was installed on the inlet line to measure the pressure within the system. 

Periosteal perfusion was performed using either a dry perfusion system (Figure 1E,F) or a wet perfusion system (Figure 1G,H). For both wet and dry perfusion the periosteum was placed on a porous platform. In the dry system the platform was raised above the level of culture medium. For wet perfusion, the platform and periosteum were submerged in the perfusate during perfusion via the periosteal artery. The tissue viability was maintained by the perfusion of the culture medium via the periosteal artery. Both systems employed silicone (internal diameter: 3.0 mm) (Gecko Optical, Perth, WA, Australia) and Tygon tubing (internal diameter: 3.175 mm) (Masterflex, Vernon Hills, IL, USA), respectively. Silicone tubing is known to be oxygen-permeable, but the perfusate oxygenation was predominately achieved by the dissolution of the atmospheric oxygen in the incubator into the perfusate [25]. Among the six periostea, periosteum 1 and 2 procured from femur were used as initial trials of our perfusion system, and we stopped the perfusion experiments at day 4. For the periosteum 3, 4, 5, and 6 procured from the scapular, we run the perfusion system for up to 4 weeks (see Table 1). 

### 2.3. Perfusate

KnockOut^TM^ Dulbecco’s modified Eagle’s medium (DMEM) supplemented with 10% KnockOut^TM^ Serum replacement and 1% antibiotic–antimycotic (100×) (Life Technologies, Grand Island, NY, USA) was used as the perfusate. Perfusate was replaced every two days until the experiments were completed. A three-way stopcock valve was installed on the bioreactor inlet to facilitate the sterile replacement of the perfusate.

### 2.4. Live/Dead Cell Analysis

Calcein AM (2 µM, Life Technologies (Eugene, OR, USA)) and ethidium homodimer-1 (EthD-1) (4 µM, Life Technologies) were used to identify live (stain green) and dead cells (stain red) within periosteal tissue biopsies, respectively, according to the manufacturer’s instructions. Briefly, biopsies were collected with sterile scissors every 2 days from the periphery of the periosteum. These sites were selected to ensure that the arterial network was not injured. The biopsies were incubated with the reagents diluted in phenol free media at 37 °C for 30 min, followed by washings with phosphate-buffered saline (Thermo Fisher Scientific). A Leica Thunder Imaging System (Leica Microsystems, Wetzlar, Germany) was used with large-volume computational clearing for real-time fluorescence imaging of the 3D tissue specimens. Images were processed using Leica LAS X advanced image analysis software and analysed using Fiji (Image J) software (version 1.53t, http://imagej.nih.gov/ij; National Institute of Mental Health, Bethesda, MD, USA). After imaging, constructs were fixed in 10% formalin for at least 48 h prior to histological analysis. Image analysis involved the merging of images from multiple optical planes per biopsy. Image processing required the separation of the green and red channels, correction for the background intensity, and image segmentation with the selection of an appropriate threshold for the subsequent automation of live or dead cell counting.

### 2.5. PrestoBlue Cell Viability and Metabolic Assay

PrestoBlue is a resazurin-based metabolic and cell viability assay. It utilizes resazurin which enters the cytoplasm and can only be reduced by viable and metabolically active cells to generate detectable fluorescence [26]. The assay was used according to the manufacturer’s instructions (Life Technologies, Eugene, OR, USA). Biopsies were collected with sterile scissors every 2 days from the periphery of the periosteum and incubated with the reagent (10% of total solution volume) in culture medium for 1 h at 37 °C. These biopsies’ sites were selected to ensure that the arterial network was not injured. Following incubation, for each sample, the supernatant (100 μL) was transferred to the wells of a 96-well plate and screened by a CLARIO star Plus microplate reader (BMG Labtech, Mornington, VIC, Australia) to read fluorescence intensity. After processing, constructs were fixed in 10% formalin for at least 48 h prior to histological analysis.

### 2.6. pH Measurement

The pH of the perfusion medium was regularly monitored as an indicator of cell metabolic activity. Perfusate was collected every 2 days and its pH was immediately determined using a benchtop pH meter (Thermo Fisher Scientific, Chelmsford, MA, USA) at room temperature. Fresh (unused) perfusate was used as a control. 

### 2.7. Histology 

Histological analysis was performed on the fixed periosteal biopsies initially collected for Live/Dead cell and PrestoBlue analyses in a clinically accredited setting. The biopsies were sectioned and transferred into tissue cassettes for processing using a Leica Peloris Tissue Processor (Leica Biosystems, Melbourne, VIC, Australia). The sections were then embedded in paraffin on a TES Valida^®^ Paraffin Embedding Centre (Medite GmbH, Wollenweberstr, Rostock, Germany), sectioned at 4 µm thickness with a Leica RM2235 Rotary Microtome (Leica Biosystems, Melbourne, VIC, Australia), and mounted on glass slides for hematoxylin and eosin staining (H&E) with Sakura Tissue-Tek Prisma^®^ Automated Slide Stainer (Sakura Fineteck USA, Inc., Torrance, CA, USA). 

## 3. Results 

**Figure 2:**
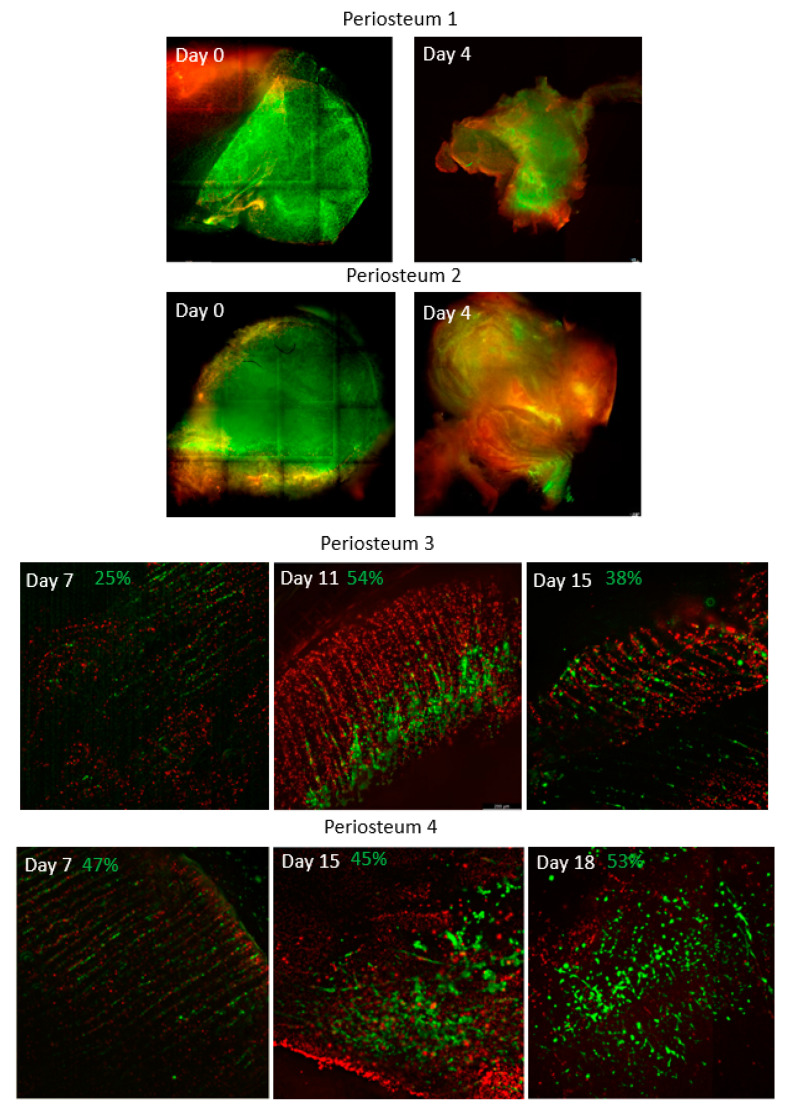

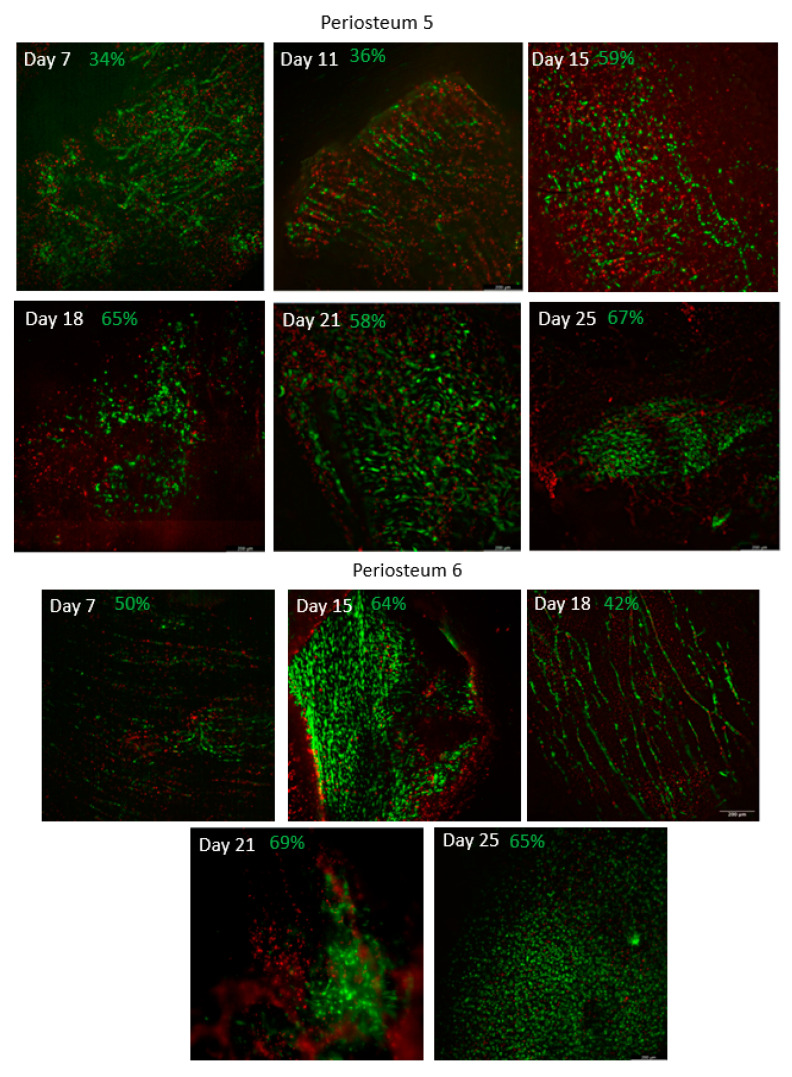
Live/dead cell analysis of biopsies collected at various time points from the 6 periosteal flaps during ex vivo perfusion. Images of periostea 1 and 2 are prepared by computational stitching (tile scanning) together with multiple images to show the large areas of the tissues. Quantitative assessments of periostea 3–6 indicate that the cell viability of periosteal tissues tended to increase with the increasing duration of perfusion and with successive trials. Live cells are indicated in green while dead cells are indicated in red.

Initially, periostea (1 and 2) were perfused for only 4 days, with succeeding studies continued for up to 25 days. Live/Dead cell analysis, PrestoBlue cell viability and metabolic assays supported the proof-of-concept for the maintenance of periosteal flaps within the bioreactor perfusion system. Firstly, measures of cell viability, using Calcein AM and Eth-D-1, ranged from 25% in the initial preliminary trials and increased to 65% following the refinement of the system. As shown in Figure 2, the proportion of live cells within the periostea increased with the longer duration of perfusion and successive trials. 

**Figure 3:**
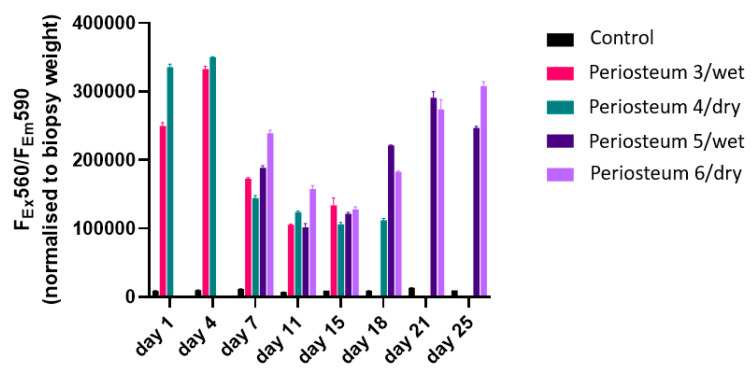
PrestoBlue-based cell viability and metabolic assay of biopsies collected at various time points from the 6 periosteal flaps during ex vivo perfusion.

Figure 3 demonstrates the PrestoBlue assays performed on periosteal biopsies collected from each perfusion system at various time points. As shown, while negligible cell viability indicators were detected in the negative control medium, it was clearly detectable for periosteum perfusates. For periosteum 3 and periosteum 4, a notable decrease was measured following day 4, with levels stabilizing from day 11. For periosteum 5 and periosteum 6, metabolic activity initially declined until day 15, but recovered thereafter with increasing activity for the duration of testing up to day 25. 

**Figure 4:**
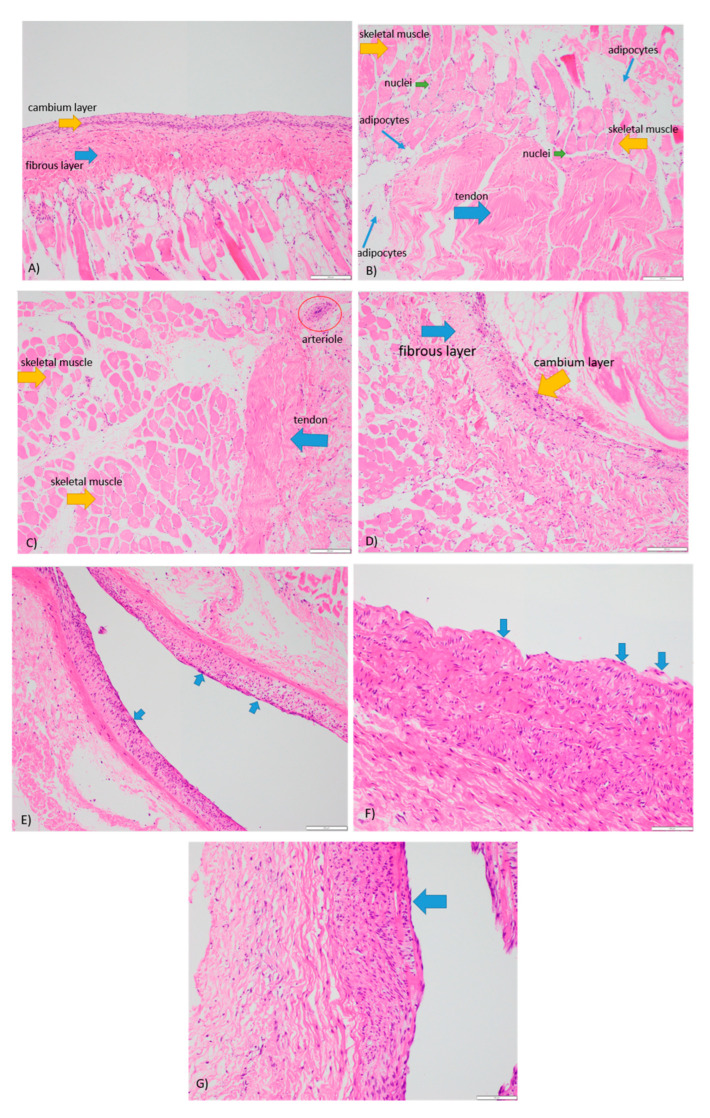
H&E staining of periostea (×40). (**A**) Periosteum 3 maintained for 17 days within a wet perfusion system. (**B**) Periosteum 4 maintained for 18 days within a dry perfusion system. (**C**) Periosteum 5 maintained for 25 days within a wet perfusion system. (**D**) Periosteum 6 maintained for 25 days within a dry perfusion system. (**E**–**G**) Viable endothelial cells (blue arrow) on periostea 4, 5, and 6, respectively.

Figure 4, as shown below, demonstrates the histological staining for periosteum 3, 4, 5, and 6, where cellular viability was assessed according to the morphology and integrity of cellular membrane and nucleus. All specimens showed appropriate amounts of muscle, fibrocollagenous tissue, fat, and periosteum with high cellular viability. Focal fat necrosis was seen in periosteum 3, 4, and 5, and focal areas of devitalized anucleate myocytes were adherent to periosteum 4 and 5. Degenerative changes were seen predominantly at the periphery of the tissue sections and most likely represent the handling artefact. Periosteum 6 exhibited the highest degree of cellular viability consistent with the Live/Dead and PrestoBlue assays. The blood vessels in all periosteum specimens were viable with preserved endothelial cells (Figure 4E–G), demonstrating the efficacy of the perfusion system to maintain the structural integrity of the blood vessels in each periosteum. Well-defined fibrous and cambium layers were observed for periosteum 3 (Figure 4A), while periosteum 6 (Figure 4D) showed the increased fragmentation of the cambium layer. Whilst we were unable to identify a clearly defined inner cambium layer in specimens 4 and 5, both showed viable adipocytes, skeletal muscles, and fibrocollagenous tissue (Figure 4B,C).

**Figure 5:**
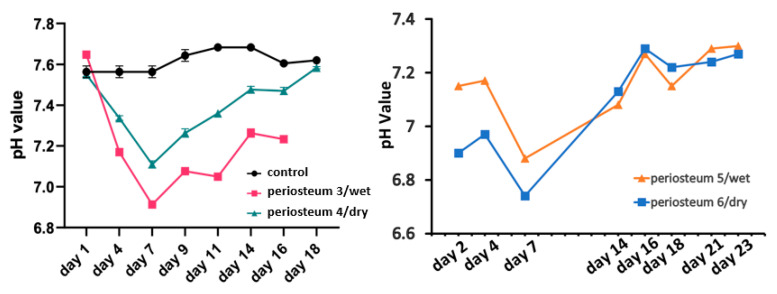
pH changes of the perfusion medium.

Figure 5 (shown below) demonstrates the changes in pH of the perfusion medium. The fresh medium was stored in the incubator as the control and pH change was buffered by 5% CO_2_ in the incubator and bicarbonate in the medium. The control pH for periosteum 3 and periosteum 4 was approximately 7.6, and for periosteum 5 and periosteum 6, the control pH was 7.4. In all four experiments, the pH declined quickly during the first 7 days, and then gradually returned to the baseline value at the end of the experiments. One mechanism which may account for the increased medium acidity from day 1 to day 7 is lactate production due to cell death [27]. After day 7, the culture medium was decreasingly consumed, and the pH value returned to that of the control medium. 

Taken together, the results support the ability of our perfusion bioreactor system to maintain the viability of a large proportion of the periosteal flaps for up to 4 weeks, with the ongoing improvement of tissue viability expected to be attained through the further optimization of the system.

## 4. Discussion

This preliminary work describes the outcomes of using a novel ex vivo perfusion bioreactor system to maintain the viability of the sheep periosteum and surrounding tissues. Six periosteal flaps were procured and cultured in 3D printed bioreactors with two different approaches: wet (emersed in the medium) or dry perfusion (separated from the medium). Tissue oxygenation was mainly achieved by the passive diffusion of atmospheric oxygen dissolved in the culture medium. The periostea were collected with adjacent (fat and muscle) tissues necessary to maintain the periosteal vascular network [28]. The results from Live/Dead and PrestoBlue assays as well as the histological staining demonstrate tissue viability for almost 4 weeks, including inner cambium layers and endothelium, as verified by H&E staining. Further investigations into appropriate surgical techniques are required to improve the cambium layer integrity during the periosteal procurement. 

Having established the proof-of-concept of our perfusion system, there is scope for further optimization, including the refining of the perfusate flow rate, perfusion pressure, and oxygenation, as well as investigating the tissue sizing and efficacy of the dry and wet perfusion. These variables are critical to recapitulating the in vivo physiological conditions for periosteal tissue and better understanding tissue utility. For example, an inappropriate flow rate and pattern were reported to affect cellular viability, interactions, and osteogenic response [29,30,31,32]. Therefore, future work will refine the system towards employing perfused periosteal tissues for augmenting in vitro bone engineering and/or in vivo clinical bone repair. In addition, we will trial the periosteum procured from different sites, especially the mandible, and evaluate their osteogenic capacities. Our intention is to use the human periosteum which will accelerate osteogenesis and vascularization. If this approach is successful, it will replace the current use of osseous free flaps in patients with critical sized bone defects.

To our knowledge, the ex vivo culture of periosteum using a perfusion bioreactor has not been reported. The outcome of the present work proves the concept of using this approach and represents an important step towards the clinical application of periosteum in scaffold vascularization for bone regeneration. 

## Figures and Tables

**Table 1:** The differences between the six experiments conducted to optimize the perfusion and preservation of periosteum are summarized. Experimental regimen for testing periosteal perfusion using our bioreactor system.

Case Number	Procurement Site (Weight)	Perfusion Mode	Tubing	Frequency of Medium Change	System Duration (Days)
1	Femur (7~8 g)	Dry perfusion	Silicone	Every two days	4
2	Femur (7~8 g)	Dry perfusion			4
3	Scapula (18~21 g)	Wet perfusion	Silicone		17
4	Scapula (18~21 g)	Dry perfusion			18
5	Scapula (18~21 g)	Wet perfusion	Tygon		25
6	Scapula (18~21 g)	Dry perfusion			25

## Data Availability

The data that support the findings of this study are available upon request from the corresponding authors.

## Abbreviations

3D printing	Three-dimensional printing
PC2 laboratory	Physical contaminant level 2 laboratory
DMEM	Dulbecco’s modified Eagle’s medium
Calcein AM	Calcein acetoxymethyl
EthD-1	Ethidium homodimer-1
H&E staining	Hematoxylin and eosin staining
